# ISG15 Connects Autophagy and IFN-γ-Dependent Control of Toxoplasma gondii Infection in Human Cells

**DOI:** 10.1128/mBio.00852-20

**Published:** 2020-10-06

**Authors:** Jaya Bhushan, Joshua B. Radke, Yi-Chieh Perng, Michael Mcallaster, Deborah J. Lenschow, Herbert W. Virgin, L. David Sibley

**Affiliations:** a Department of Molecular Microbiology, Washington University School of Medicine, Saint Louis, Missouri, USA; b Department of Medicine, Washington University School of Medicine, Saint Louis, Missouri, USA; c Department of Pathology and Immunology, Washington University School of Medicine, Saint Louis, Missouri, USA; Albert Einstein College of Medicine

**Keywords:** ATG5, BioID, ubiquitin, autophagy adaptors, LC3, ISGylation, parasitophorous vacuole, parasite, autophagy, intracellular parasites, ubiquitination

## Abstract

The intracellular protozoan parasite Toxoplasma gondii is capable of infecting most nucleated cells, where it survives in a specially modified compartment called the parasitophorous vacuole (PV). Interferon gamma (IFN-γ) is the major cytokine involved in activating cell-autonomous immune responses to inhibit parasite growth within this intracellular niche. In HeLa cells, IFN-γ treatment leads to ubiquitination of susceptible parasite strains, recruitment of the adaptors p62 and NDP52, and engulfment in microtubule-associated protein 1 light chain 3 (LC3)-positive membranes that restrict parasite growth. IFN-γ-mediated growth restriction depends on core members of the autophagy (ATG) pathway but not the initiation or degradative steps in the process. To explore the connection between these different pathways, we used permissive biotin ligation to identify proteins that interact with ATG5 in an IFN-γ-dependent fashion. Network analysis of the ATG5 interactome identified interferon-stimulated gene 15 (ISG15), which is highly upregulated by IFN treatment, as a hub connecting the ATG complex with other IFN-γ-induced genes, suggesting that it forms a functional link between the pathways. Deletion of ISG15 resulted in impaired recruitment of p62, NDP52, and LC3 to the PV and loss of IFN-γ-restricted parasite growth. The function of ISG15 required conjugation, and a number of ISGylated targets overlapped with the IFN-γ-dependent ATG5 interactome, including the adapter p62. Collectively, our findings establish a role for ISG15 in connecting the ATG pathway with IFN-γ-dependent restriction of T. gondii in human cells.

## Abstract (precis)

Interferon(s) provide the primary defense against intracellular pathogens, a property ascribed to their ability to upregulate interferon-stimulated genes. Due to the sequestered niche occupied by Toxoplasma gondii, the host has elaborated intricate ways to target the parasite within its vacuole. One such mechanism is the recognition by a noncanonical autophagy pathway that envelops the parasite-containing vacuole and stunts growth in human cells. Remarkably, autophagy-dependent growth restriction requires interferon-γ, yet none of the classical components of autophagy are induced by interferon. Our studies draw a connection between these pathways by demonstrating that the antiviral protein ISG15, which is normally upregulated by interferons, links the autophagy-mediated control to ubiquitination of the vacuole. These findings suggest a similar link between interferon-γ signaling and autophagy that may underlie defense against other intracellular pathogens.

## INTRODUCTION

The apicomplexan Toxoplasma gondii is a common parasite of warm-blooded animals that also causes zoonotic infections in humans, most importantly due to congenital transmission or opportunistic infections in immunocompromised patients (1). The parasite resides within the parasitophorous vacuole (PV), which is formed by invagination of the plasma membrane during active invasion of the parasite, and this unique intracellular niche is critical to avoidance of lysosomal fusion and survival (2). Strains of T. gondii comprise three predominant lineages that are common in North America and Europe (3). Type I strains are rare in humans but highly virulent in mice, type II strains are of intermediate virulence in mice and the most common in human infection, and type III strains are avirulent in mice and common in domestic animals but very rare in humans (3). Control of T. gondii infection is dependent on interferon gamma (IFN-γ) signaling in both murine (4, 5) and human cells (6, 7), although the mechanisms differ between species and cell types.

In murine cells, stimulation with IFN-γ results in upregulation of a variety of interferon-stimulated genes (ISGs), including immunity-related GTPases (IRGs) and guanylate binding proteins (GBPs) that play important roles in host defense (8, 9). Recruitment of IRGs (10, 11) and GBPs (12–14) to PVs surrounding susceptible parasite strains results in vesiculation of the PV membrane and destruction of the parasite. Type I strains, which are highly virulent in laboratory mice, largely avoid this fate, while types II and III are highly susceptible (15). These pathways are counteracted by secretion of a parasite family of polymorphic ROP kinases that target IRGs and GBPs to prevent their accumulation on the PV, thus protecting this intracellular niche (15, 16). The IRG family has undergone expansion and selection for polymorphism in mice, where they play a major role in host defense; however, they are largely absent in human cells (17). As such, human cells rely on overlapping and distinct pathways for IFN-γ-dependent control of parasite replication, including depletion of tryptophan via indoleamine 2,3-dioxygenase, select GBPs, generation of reactive oxygen species, ubiquitination, and lysosomal clearance (18).

One IFN-γ-dependent control mechanism that is shared between murine and human cells is involvement of proteins in the autophagy (ATG) pathway, which is normally involved in cellular remodeling and nutrient recycling but also plays important roles in host defense (19). Core components of the ATG pathway that are involved in lipidation and recruitment of microtubule-associated protein 1 light chain 3 (LC3) are required for control of T. gondii infection in IFN-γ-treated murine and human cells (18). Key members of this pathway include the ATG5-12-16L1 complex, as well as ATG3 and ATG7, which are required for recruitment of IRGs and vesiculation of the PV in murine cells activated with IFN-γ (20, 21). This complex of ATG proteins may be directly involved in the recruitment of IRGs to the PV (20) or, alternatively, be required for homeostasis of the IRG system (21). The upstream initiation steps (i.e., ATG14 and Beclin) and downstream degradative steps (i.e., lysosome fusion) of the ATG pathway are not required for control of T. gondii infection in murine cells (20, 21). IFN-γ-mediated control of T. gondii in human HeLa cells requires a similar complex of ATG proteins, including ATG5 and ATG7 (22). However, unlike the murine system, engagement of this pathway does not lead to PV vesiculation in human cells. Rather, recruitment of LC3 to PVs containing susceptible strains of T. gondii results in formation of multiple membranes around PV and stunted parasite growth (22). Similar to murine cells, type I strains are largely resistant to ATG-dependent growth inhibition, while types II and III are susceptible to this pathway (22). The first step in this pathway is ubiquitination (Ub) of the PV, followed by recruitment of adaptors p62 and NDP52 and decoration by LC3B (22). Similar to the murine system, this process requires ATG16L1 and ATG7 but is independent of ATG14 and Beclin (22). In this regard, the ATG-dependent pathway for restriction of T. gondii growth in human cells resembles xenophagy (23), with the exceptions that the former is IFN-γ dependent and it does not lead to lysosomal fusion in HeLa cells. Separately, it was described that IFN-γ activation of HUVEC cells, a human endothelial cell line, leads to ubiquitination, recruitment of p62 and NDP52, fusion with lysosomes, and destruction in a process that is not dependent on ATG16L1. This difference in reliance on ATG proteins likely reflects differences in the mechanisms of cell-autonomous immunity between different human cell types (24).

IFN-γ signaling activates cell-autonomous immunity by upregulating interferon-stimulated genes (ISGs) to counteract intracellular pathogens (9, 25, 26). Although many genes are upregulated by IFN-γ, ATG proteins are not typically among them. Hence, the link between the IFN-γ-dependent cell-autonomous control of pathogens and ATG pathways is unclear. IFN-γ upregulates the expression of another ubiquitin-ligase-related system in cells that depends on the protein interferon-stimulated gene 15 (ISG15) (27). ISG15 forms covalent and noncovalent interactions with host cellular proteins and governs diverse cellular pathways, including host defense and autophagy (28). Previously published studies have largely focused on type I IFN-dependent protein ISGylation during viral infection (29). However, a recent study has shown that free ISG15 is important for recruitment of interleukin-1β (IL-1β)-producing dendritic cells at the site of T. gondii infection in mice (30). The role of ISG15 has not been examined in human cells infected with T. gondii.

Here, we were interested in identifying the connection between the ATG pathway and IFN-γ signaling that leads to control of T. gondii infection in human cells. We found that ISG15 functioned by governing IFN-γ-dependent recruitment of ATG mediators to PVs occupied by intracellular T. gondii. Hence, ISG15 forms an important molecular link between the ATG and IFN-γ signaling pathways to enable cell-autonomous host defense in human cells.

## RESULTS

### Identification of IFN-γ-dependent interactors of ATG5 by proximity-based biotin labeling.

**FIG 1:**
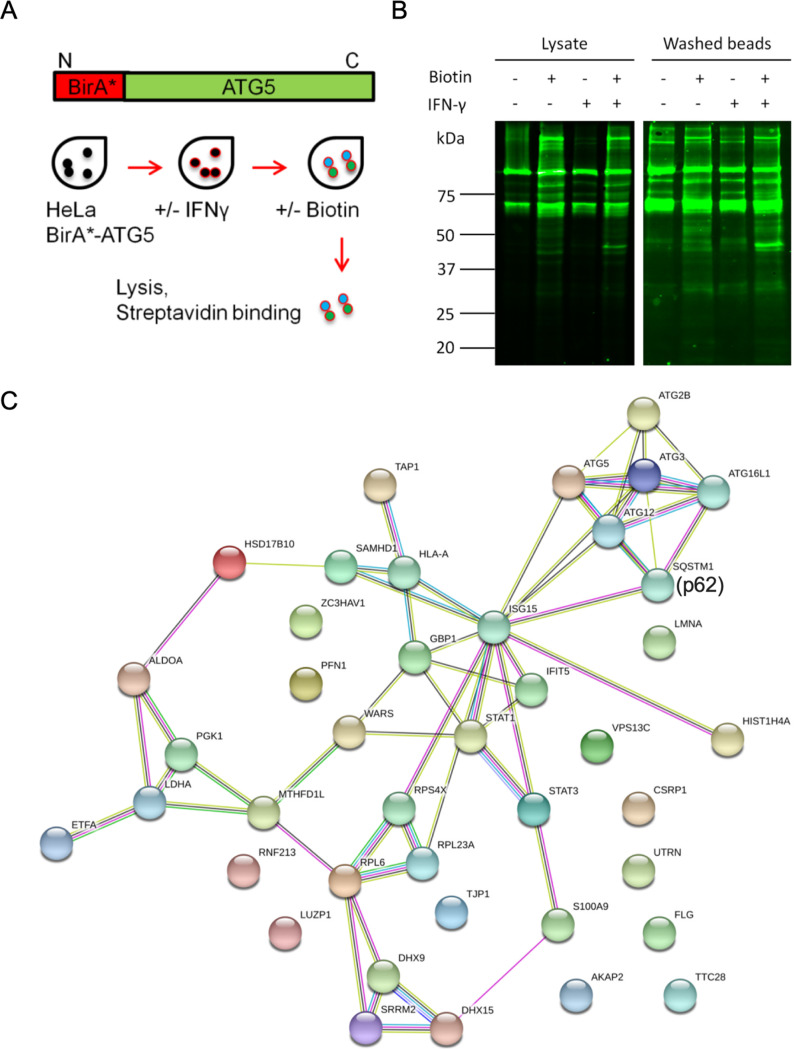
Identification of ATG5 interacting proteins by permissive biotin ligase labeling. (A) Experimental design for identification of ATG5 interactors by proximity-based biotin labeling. HeLa cells expressing an N-terminal fusion with permissive biotin ligase BirA were treated with or without IFN-γ (100 U/ml) for 24 h. Following stimulation, cells were grown in the presence of biotin (150 μM) for 18 h, lysed in detergent, and affinity purified using streptavidin beads. (B) Biotinylated proteins in cell lysates and washed beads from the experimental set up in panel A were detected by Western blotting with IRDye 800CW-labeled (green) streptavidin. The lysate blot was imaged at intensity 4, and the washed bead blot was imaged at intensity 7. (C) Network of the ATG5 interactome using the STRING database. Proteins identified by mass spectrometry were analyzed using Scaffold version 4.0. The normalized weighted spectra of IFN-γ-stimulated samples were compared with unstimulated samples that were both grown in the presence of biotin. Proteins showing enrichment (see Table 1) are represented by nodes (filled circles) in the network, and the edges indicate interactions. Criteria for interactions are shown by colored lines as follows: red line, gene fusion events; green line, gene neighborhood; blue line, gene cooccurrence; purple line, experimental evidence; yellow line, text mining; light blue line, database; black line, coexpression.

To explore the role of ATG in IFN-mediated control of intracellular parasite growth, we examined proteins that interact with ATG5 in an IFN-γ-dependent fashion using proximity-based biotin labeling. For this purpose, we generated a stable line of HeLa cells expressing ATG5 with the permissive biotin ligase BirA (31) fused to its N terminus, referred to as BirA-ATG5. We stimulated HeLa cells expressing BirA-ATG5 with IFN-γ followed by addition of biotin in the medium and affinity purification of the biotinylated proteins using streptavidin beads (Fig. 1A). Immunoblotting of the immunoprecipitated proteins with streptavidin revealed a number of endogenous proteins that were labeled in the absence of added biotin, consistent with previous reports of host proteins that are normally biotinylated in human cells (32) (Fig. 1B).

We next compared proteomic profiles generated by label-free mass spectrometry of IFN-γ-stimulated samples with unstimulated samples, grown in the presence of biotin. Samples were repeated in four separate experiments, and the combined data were used to analyze the interactome of ATG5. In order to identify IFN-γ-dependent ATG5 interactors, we selected proteins on the basis of peptide count (≥2 peptides in the average of 4 replicates) and fold change in normalized weighted spectra (≥2 in IFN-γ-stimulated versus unstimulated control samples) (Table 1). We also included the autophagy proteins (SQSTM1, ATG12, ATG16L1, ATG3, and ATG2B), as they are essential for the autophagy pathway (19). The final IFN-γ-dependent ATG5 interactome comprised 40 proteins (Table 1). We identified 87 interactors of ATG5 in naive HeLa cells and 40 interactors that were enhanced in IFN-γ-stimulated HeLa cells using permissive biotin labeling. Previously, Behrends et al. (33) identified 142 interactors of HA-tagged ATG5 by immunoprecipitation in HEK293T cells; however, they only considered four of these to be high-confidence interactors. Comparison of the 4 high-confidence interactors (i.e., ATG16L1, TECPR1, TKT, and ATG12) showed that 3 of these proteins (i.e., ATG16L1, ATG12, and TECPR1) overlapped with our list of ATG5 interactors in naive cells, and 2 (i.e., ATG16L1 and ATG12) of these overlapped with our list of IFN-γ-stimulated interactors in HeLa cells. The differences in proteins detected by these two methods likely represent a combination of cell types, experimental approaches, and criteria for classifying meaningful interactions.

**TABLE 1:** IFN-γ-dependent ATG5 interactome identified by proximity labeling

Identified proteins	Gene name	Fold change^*a*^	Naive replicates^*b*^				IFN-γ-stimulated replicates^*b*^			
Tryptophan-tRNA ligase, cytoplasmic	WARS	24	4	6	2	1	67	91	85	72
Cluster of signal transducer and activator of transcription 1-alpha/beta	STAT1	15	4	8	1	1	54	87	22	46
Ubiquitin-like protein ISG15	ISG15	6.6	1	1	1	1	13	8	1	4
E3 ubiquitin-protein ligase RNF213	RNF213	4.1	1	1	1	1	3	9	1	3
3-hydroxyacyl-CoA dehydrogenase type-2	HSD17B10	4	1	1	1	1	1	1	1	13
Antigen peptide transporter 1	TAP1	3.7	1	1	1	1	5	5	3	2
Cluster of HLA class I histocompatibility antigen, A-68 alpha chain	HLA-A	3.7	1	3	1	1	4	11	4	4
Filaggrin	FLG	3.6	4	3	1	3	10	2	21	11
Histone H4	HIST1H4A	3.3	1	1	4	2	1	1	14	9
Protein S100-A9	S100A9	3.3	1	1	4	6	15	1	4	20
Phosphoglycerate kinase 1	PGK1	3	1	3	3	1	1	15	5	4
Zinc finger CCCH-type antiviral protein 1	ZC3HAV1	3	1	2	1	1	3	8	1	4
Tight junction protein ZO-1	TJP1	2.9	1	5	1	2	5	14	1	7
Cluster of guanylate-binding protein 1	GBP1	2.9	1	1	1	1	1	6	1	4
Interferon-induced protein with tetratricopeptide repeats 5	IFIT5	2.7	1	1	1	1	2	6	1	2
Electron transfer flavoprotein subunit alpha, mitochondrial	ETFA	2.6	1	1	1	2	1	2	1	9
Serine/arginine repetitive matrix protein 2	SRRM2	2.6	1	1	1	1	2	8	1	1
Profilin-1	PFN1	2.5	1	3	1	1	2	12	1	3
Monofunctional C1-tetrahydrofolate synthase, mitochondrial	MTHFD1L	2.4	1	1	1	1	1	1	1	7
Deoxynucleoside triphosphate triphosphohydrolase SAMHD1	SAMHD1	2.3	1	1	1	1	1	6	1	1
Pre-mRNA-splicing factor ATP-dependent RNA helicase DHX15	DHX15	2.2	3	1	1	1	7	5	1	1
Cysteine and glycine-rich protein 1	CSRP1	2.2	4	2	2	2	8	5	4	6
Leucine zipper protein 1	LUZP1	2.2	1	3	1	1	5	8	1	1
Vacuolar protein sorting-associated protein 13C	VPS13C	2.1	9	17	1	1	18	28	1	11
60S ribosomal protein L6	RPL6	2.1	3	1	3	1	1	6	6	4
60S ribosomal protein L23a	RPL23A	2.1	3	2	1	1	3	5	1	6
Signal transducer and activator of transcription 3	STAT3	2.1	1	7	1	1	8	11	1	2
A-kinase anchor protein 2	AKAP2	2.2	1	3	1	1	3	9	1	1
40S ribosomal protein S4, X isoform	RPS4X	2.1	3	1	1	1	8	2	1	1
ATP-dependent RNA helicase A	DHX9	2.2	1	3	1	1	1	8	1	4
Utrophin	UTRN	2	39	42	6	11	70	81	15	30
l-lactate dehydrogenase A chain	LDHA	2	4	10	4	2	2	25	5	9
Prelamin-A/C	LMNA	2	1	1	5	3	1	6	8	5
Fructose-bisphosphate aldolase A	ALDOA	2	1	2	5	1	1	7	8	2
Tetratricopeptide repeat protein 28	TTC28	2	1	3	1	1	2	9	1	1
Sequestosome-1	SQSTM1	1.4	13	5	2	2	18	7	3	1
Ubiquitin-like protein ATG12	ATG12	1.3	7	3	2	1	8	4	1	4
Autophagy-related protein 16-1	ATG16L1	1	19	17	6	12	18	19	10	8
Ubiquitin-like-conjugating enzyme ATG3	ATG3	0.9	4	5	2	4	3	5	1	4
Autophagy-related protein 2 homolog B	ATG2B	0.8	1	2	1	1	1	1	1	1

a Fold change in normalized weighted spectra in IFN-γ-stimulated versus unstimulated samples. The criteria for inclusion are (i) ≥2 peptides in the average of 4 replicates and (ii) ≥2-fold increase in IFN-γ-stimulated versus unstimulated control samples.

b Replicates wherein columns represent normalized weighted spectra.

To visualize the interactome, we analyzed the resulting list using the STRING database (version 10.5), which builds protein-protein interactions based on experimental evidence and predictions based on genomic context (34). As expected, the ATG proteins, including the adaptor SQSTM1 (also known as p62), which is responsible for cargo recognition, and components involved in autophagosome formation and closure (i.e., ATG2B, ATG3, ATG5, ATG12, and ATG16L1) (35, 36), formed a tightly interacting cluster (Fig. 1C). ISG15 formed a central hub in the network (Fig. 1C). ISG15 showed high fold change with IFN-γ treatment (6.6-fold increase) and interacted directly with ATG5, ATG12, ATG3, and SQSTM1 (evidence from text mining and experimental data) (Fig. 1C) (37). Based on multiple lines of evidence, ISG15 also makes connections with the proteins GBP1, STAT1, and STAT3, which are known to play important roles during T. gondii infection (Fig. 1C) (38). The STAT1 cluster also included WARS and IFTI5, which are known to be upregulated by IFN-γ (39, 40) and augment antiviral host defense (41, 42) (Fig. 1B). ISG15 also interacted with SAMHD1, which is induced by IFNs and has antiviral function (43). S100A9, which is known to be upregulated during infection and inflammation (44), was also present in the ATG5 interactome. IFN-γ is associated with metabolic reprogramming, and enzymes of the glycolytic pathway (i.e., PGK1, ALDOA, and LDHA) formed a separate cluster of the ATG5 interactome (Table 1, Fig. 1C) (45). The ribosomal protein cluster consisting of RPS4X, RPL23A, and RPL6 interacted with other protein clusters via ISG15 and also with a cluster enriched in proteins implicated in viral RNA sensing, DHX9 and DHX15 (46) (Fig. 1C).The network also contained several mitochondrial proteins (i.e., ETFA, HSD17B10, and MTHFD1L) and transport proteins such as VPS13C, which has been implicated in mitophagy (47). Based on the central position of ISG15 in the ATG5 interactome, we decided to focus on this protein, which plays important roles in antiviral defense (48) but has not previously been implicated in cell-autonomous control of T. gondii.

### IFN-γ stimulation induces ISG15 expression and ISGylation in human cells.

**FIG 2:**
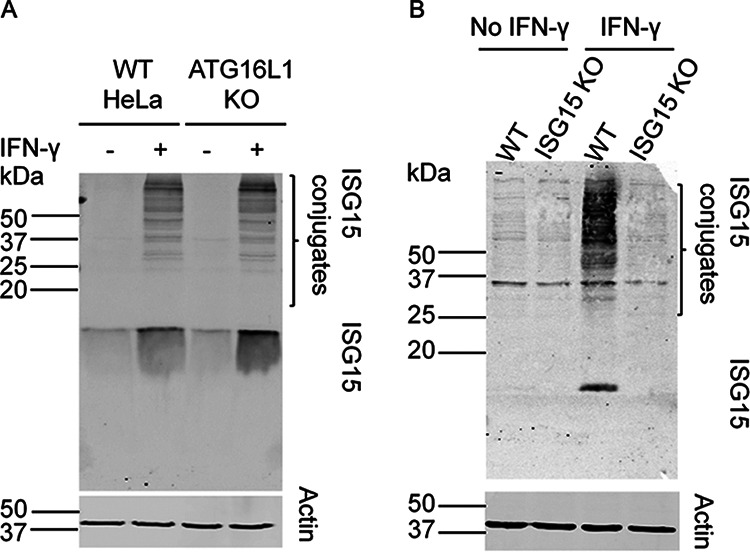
IFN-γ stimulation induces ISG15 expression and ISGylation in human cells. (A) Wild-type (WT) and ATG16L1 knockout (KO) HeLa cells were grown in ± IFN-γ (100 U/ml) for 24 h. Cell lysates were prepared and equal amounts of protein were loaded for immunoblotting. ISG15 expression (∼17 kDa) and protein ISGylation (higher-molecular-weight bands) were detected using rabbit polyclonal ISG15 antibody and LI-COR IRDye 800CW (green) goat anti-rabbit IgG. As a loading control, a parallel blot was probed with mouse monoclonal actin antibody (43 kDa) and LI-COR IRDye 680CW (red) goat anti-mouse IgG. (B) Wild-type (WT) and ISG15 knockout (KO) A549 cells were grown in ± IFN-γ (100 U/ml) for 24 h. Cell lysates were prepared and equal amounts of protein were loaded for immunoblotting. ISG15 expression and protein ISGylation were detected using rabbit polyclonal ISG15 antibody and LI-COR IRDye 800CW (green) goat anti-rabbit IgG. As a loading control a parallel blot was probed with mouse monoclonal actin antibody and LI-COR IRDye 800CW (green) goat anti-mouse IgG. Both wild-type and ISG15 KO cells showed low levels of cross-reactivity to anti-ISG15 by Western blotting (i.e., band around 35 kDa).

We observed a high fold change for interaction of ATG5 with ISG15 in IFN-γ-treated samples (Table 1). This prompted us to study the effect of IFN-γ on ISG15 induction and ISGylation of cellular proteins in HeLa (cervical) and A549 (lung) cells. Both cell types are human epithelial in origin, and as shown below, the ATG-dependent control of T. gondii growth previously described in HeLA cells also extends to A549 cells. We observed that in the absence of IFN-γ, HeLa cells expressed low levels of ISG15, and stimulation with IFN-γ enhanced ISG15 levels (Fig. 2A). Addition of IFN-γ also led to ISGylation of cellular proteins that were detected as a high-molecular-weight smear, while these high-molecular-weight ISG15 conjugates were absent in unstimulated samples (Fig. 2A). The ATG5 interactome showed an interaction between ISG15 and the ATG protein cluster, suggesting that ATG proteins may affect ISG15 expression and ISGylation. To examine the role of ATG in IFN-γ-dependent ISG15 expression and conjugate formation, we used HeLa cells lacking the key ATG protein ATG16L1 (Fig. 2A). We observed that the absence of ATG16L1 in HeLa cells did not affect IFN-γ-dependent ISG15 expression or ISGylation of cellular proteins (Fig. 2A). We also observed a similar upregulation of ISG15 and ISGylation in A549 cells in response to IFN-γ treatment (Fig. 2B).

### ISG15 does not impact ubiquitination of the PV but is important for recruitment of p62, NDP52, and LC3.

**FIG 3:**
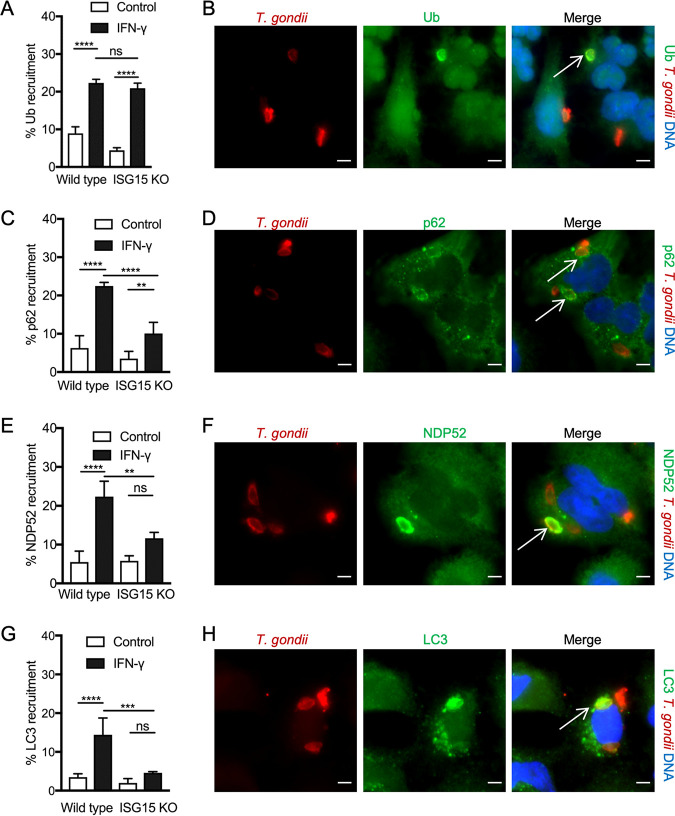
Role of ISG15 in IFN-γ dependent recruitment of autophagy mediators. Wild-type (WT) and ISG15 knockout (KO) A549 cells were grown with or without IFN-γ (100 U/ml) for 24 h. Cells were infected with type III T. gondii CTG strain tachyzoites and washed 2 h postinfection. (A to H) Recruitment of autophagy mediators as indicated by percentage of PV positive for (A and B) ubiquitin (Ub), (C and D) p62, (E and F) NDP52, and (G and H) LC3 was determined 6 h postinfection by immunofluorescence microscopy. The values represent means ± standard error from three independent experiments, each containing three internal replicates. Two-way ANOVA was used to determine statistical significance. *P* values of ≤0.05 (*), ≤0.01 (**), ≤0.001 (***), and ≤0.0001 (****) were considered statistically significant. ns, not significant. Representative images showing recruitment are on the right. Arrows indicate positive recruitment. Scale bar = 5 μm. Parasites (red) were identified by SAG1 (mouse monoclonal DG52 antibody) or tachyzoite (rabbit polyclonal anti-whole tachyzoite antibody) staining, followed by secondary antibodies conjugated to Alexa Fluor 594 (red) conjugated goat anti-mouse IgG or goat anti-rabbit IgG. Autophagy mediators (green) were stained with mouse monoclonal ubiquitin antibody, guinea pig polyclonal p62 antibody, rabbit polyclonal NDP52 antibody, rabbit polyclonal LC3 antibody followed by Alexa Fluor 488 (green) conjugated goat anti mouse IgG, guinea pig IgG, or rabbit IgG. Nuclei were stained with DAPI (4′,6-diamidino-2-phenylindole) (blue).

To examine the role of ISG15 in ATG-dependent control of T. gondii in IFN-γ treated cells, we examined ubiquitination and recruitment of autophagy mediators (i.e., p62, NDP52, and LC3) to the PV. Given the similar response of HeLa and A549 cells to IFN-γ, to ISG15 induction, and to protein ISGylation, we took advantage of existing wild-type and knockout (KO) lines of the human lung carcinoma epithelial line A549 (Fig. 2A and B). Similar to our previous findings with HeLa cells, IFN-γ treatment led to a significant increase in the percentage of PV that were positive for ubiquitin (Ub) (Fig. 3A and B). The increase in ubiquitin-positive cells was similar in ISG15 KO cells, indicating that this first step does not rely on ISG15 (Fig. 3A and B). Similar to previous findings in HeLa cells, recruitment of p62 to the PV in A549 cells was also enhanced in the presence of IFN-γ (Fig. 3C and D). However, in the absence of ISG15, there were significantly fewer p62-positive vacuoles compared to the wild-type cells (Fig. 3C and D). We observed a similar pattern of decreased localization of the adaptor NDP52 in ISG15 KO cells compared with wild-type cells stimulated with IFN-γ (Fig. 3E and F). As the adaptor proteins are responsible for directing the PV to LC3-positive autophagic membrane, we further quantified and compared the percentage of PV positive for LC3 between the two cell types (Fig. 3G and H). The number of LC3-positive PV increased significantly in wild-type cells in the presence of IFN-γ (Fig. 3G and H). In ISG15 KO cells, the increase was not significant compared to the unstimulated control sample. The recruitment of LC3 to PV was markedly abrogated in IFN-γ-stimulated ISG15 KO cells compared to wild-type IFN-γ-stimulated cells (Fig. 3G and H). Costaining of ubiquitin and adaptor molecules verified that the recruitment is sequential in A549 cells, similar to previous reports in HeLa cells (22). This conclusion is based on the observation that there are appreciable numbers of singly ubiquitin-positive vacuoles but very few that recruit adaptors without also being ubiquitin positive, while the majority of vacuoles are positive for both markers (Fig. S1). Moreover, in the absence of ISG15, there was an increase in singly ubiquitin-positive vacuoles with a corresponding decrease in doubly positive vacuoles staining both with ubiquitin and with each of the respective adaptors (Fig. S1). These data suggest that ISG15 does not play a role in ubiquitination of PV but is important for downstream recruitment of adaptors and LC3 to the PV.

10.1128/mBio.00852-20.1 FIG S1 Colocalization of autophagy mediators in the parasitophorous vacuole membrane. Wild-type (WT) and ISG15 knockout (KO) A549 cells were grown in the presence of IFN-γ (100 U/ml) for 24 h. Cells were infected with type III T. gondii strain CTG tachyzoites (expressing GFP) and washed 2 h postinfection. Vacuoles that were singly positive for autophagy mediators ubiquitin (Ub), p62, NDP52, and LC3 versus doubly positive for Ub and p62 or NDP52 or LC3 were quantified 6 h postinfection by immunofluorescence microscopy. The values represent means ± standard deviation containing three internal replicates. (A, B, D, E, G, H) The data are plotted in two ways, first to show the percentages of singly and doubly positive vacuoles among all cells (A, D, G) and then to express the percentages of singly and doubly positive vacuoles among those that were positive for at least one marker (B, E, H). Two-way ANOVA was used to determine statistical significance. *P* values of ≤0.05 (*), ≤0.01 (**), ≤0.001 (***), and ≤0.0001 (****) were considered statistically significant. ns, not significant. (C, F, I) Representative images showing localization of ubiquitin (Ub), p62, NDP52, and LC3 at the parasitophorous vacuole membrane. Arrowheads represent singly positive vacuoles, and arrows indicate doubly positive vacuoles. Scale bar = 5 μm. Parasites (green) were identified by expression of green fluorescent protein (GFP). Ubiquitin (Ub) (magenta or red) was stained with mouse monoclonal ubiquitin antibody, p62 (red) was stained with guinea pig polyclonal p62 antibody, NDP52 (magenta) was stained with rabbit polyclonal NDP52 antibody, and LC3 (magenta) was stained with rabbit polyclonal LC3 antibody followed by Alexa Fluor 647 (magenta) conjugated goat anti-mouse IgG or goat anti-rabbit IgG or Alexa Fluor 594 (red) conjugated goat anti-guinea pig IgG or Alexa Fluor 555 (red) conjugated goat anti-mouse IgG. Nuclei were stained with DAPI (blue). Download FIG S1, TIF file, 1.0 MB. Copyright © 2020 Bhushan et al. 2020 Bhushan et al. This content is distributed under the terms of the Creative Commons Attribution 4.0 International license.

### ISG15-deficient cells are not compromised in autophagy.

**FIG 4:**
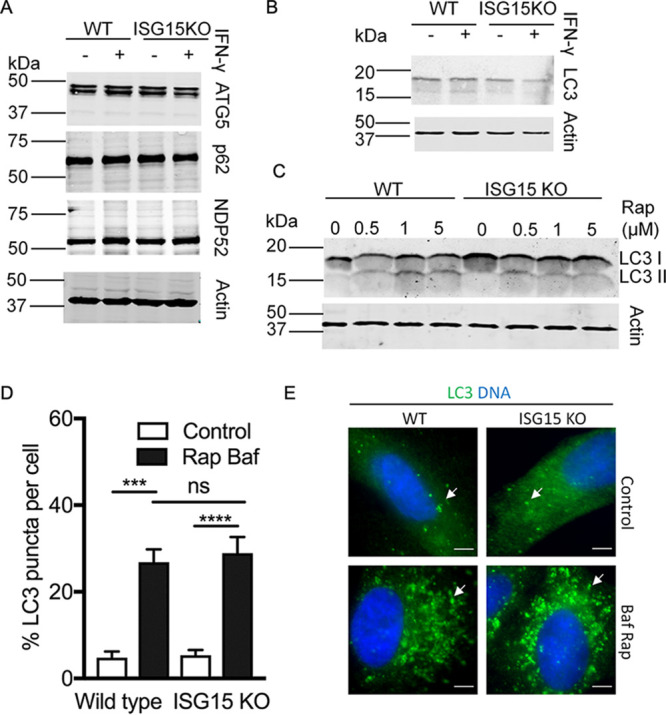
Function of macroautophagy in wild-type (WT) and ISG15 knockout (KO) cells. (A) WT and ISG15 KO A549 cells were cultured with or without IFN-γ (100 U/ml) for 24 h. Cell lysates were prepared, and equal amounts of protein were loaded for immunoblotting. The expression of autophagy proteins ATG5, p62, and NDP52 was determined by immunoblotting with antigen-specific antibodies. A parallel blot was probed for actin as the loading control. LI-COR IRDye 800CW (green) goat anti-rabbit (ATG5 and NDP52 blot) or goat anti-mouse (actin blot) IgG or LI-COR IRDye 680RD (red) goat anti-guinea pig (p62 blot) IgG were used as secondary antibodies for detection. (B) LC3 levels were compared by immunoblotting cell lysates from wild-type (WT) and ISG15 KO A549 cells grown in ± IFN-γ (100 U/ml) for 24 h using rabbit polyclonal LC3 as the primary antibody and LI-COR IRDye 800CW (green) goat anti-rabbit IgG as the secondary antibody. The same blot was reprobed for actin using mouse monoclonal actin antibody followed by LI-COR IRDye 680RD (red) goat anti-mouse IgG as the loading control. (C) Induction of autophagy was studied in wild-type (WT) and ISG15 KO A549 cells by comparing LC3 II levels of cells grown for 24 h in the presence of different concentrations of rapamycin (Rap). LI-COR IRDye 800CW (green) goat anti-rabbit IgG was used as the secondary antibody. A parallel blot was probed with mouse monoclonal actin antibody followed by LI-COR IRDye 680RD (red) goat anti-mouse IgG. (D and E) Autophagosome formation was studied in wild-type (WT) and ISG15 KO A549 cells with or without bafilomycin A1 (Baf) and rapamycin (Rap). Representative images are shown on the right. Scale bar = 5 μm. Autophagosomes were stained with polyclonal rabbit LC3 antibody. Alexa Fluor 488 (green) conjugated rabbit IgG antibody was used as the secondary antibody. Examples of puncta are indicated with white arrows.

We considered that the impaired localization of p62, NDP52, and LC3 to the PV in the absence of ISG15 could be due to defects in expression or reduced flux through the ATG pathway. To examine these possibilities, we stimulated wild-type and ISG15 KO cells with IFN-γ for 24 h and compared the levels of p62, NDP52, ATG5, and LC3 by Western blotting. We observed that the expression levels of these proteins were comparable between wild-type and ISG15 KO cells under resting conditions (Fig. 4A and B). Moreover, stimulation with IFN-γ did not affect the levels of ATG5, p62, NPD52, and LC3 proteins in either cell type (Fig. 4A and B). Infection by T. gondii, with or without IFN-γ treatment, also did not alter the levels of these ATG proteins in A549 cells (Fig. S2). We next examined the induction of autophagy by rapamycin (Rap) based on the conversion of LC3 I to LC3 II, the lipid-conjugated and membrane-associated forms (49). Conversion to the LC3 II form was similar in both wild-type and ISG15 KO cells treated with rapamycin, as detected by Western blotting (Fig. 4C). We compared autophagosome formation by enumerating LC3 puncta per cell in the presence of rapamycin (to induce autophagy) and bafilomycin A1 (to block lysosomal degradation). Addition of rapamycin (Rap) and bafilomycin A1 (Baf) led to increased LC3 puncta per cell in both wild-type and ISG15 KO cells (Fig. 4D and E). The puncta per cell were quantified, and they were comparable between wild-type and ISG15 KO cells in the presence of ATG modulators (Fig. 4D). We conclude from these experiments that ISG15 KO A549 cells are not globally impaired in autophagy.

10.1128/mBio.00852-20.2 FIG S2 Expression levels of autophagy proteins in wild-type A549 cells. A549 cells were cultured with or without IFN-γ (100 U/ml) for 24 h, followed by T. gondii infection for 24 h. Control cells were incubated in the absence of infection. Cell lysates were prepared, and equal amounts of protein were loaded for immunoblotting. (A) The expression level of ATG5 was determined by immunoblotting with antigen-specific antibody. LI-COR IRDye 800CW (green) goat anti-rabbit was used as secondary antibody for detection. The same blot was reprobed for actin using mouse monoclonal actin antibody followed by LI-COR IRDye 680RD (red) goat anti-mouse IgG as the loading control. (B) p62 and LC3 levels were compared by immunoblotting cell lysates with guinea pig polyclonal p62 and rabbit polyclonal LC3 as the primary antibody, respectively, on the same blot. LI-COR IRDye 680RD (red) goat anti-guinea pig IgG and LI-COR IRDye 800CW (green) anti-rabbit IgG were used as secondary antibodies. The same blot was reprobed for actin using mouse monoclonal actin antibody followed by LI-COR IRDye 680RD (red) goat anti-mouse IgG as the loading control. (C) The expression level of NDP52 was determined by immunoblotting with polyclonal rabbit NDP52 antibody. LI-COR IRDye 800CW (green) goat anti-rabbit IgG was used as the secondary antibody for detection. The same blot was reprobed for actin using mouse monoclonal actin antibody as the loading control, followed by LI-COR IRDye 680RD (red) goat anti-mouse IgG as the secondary antibody. (D) T. gondii infection was confirmed by immunoblotting lysates with polyclonal rabbit T. gondii aldolase as the primary antibody. LI-COR IRDye 800CW (green) goat anti-rabbit IgG was used as the secondary antibody for detection. Download FIG S2, TIF file, 0.2 MB. Copyright © 2020 Bhushan et al. 2020 Bhushan et al. This content is distributed under the terms of the Creative Commons Attribution 4.0 International license.

### ISG15 is important for IFN-γ-dependent restriction of T. gondii in human cells.

**FIG 5:**
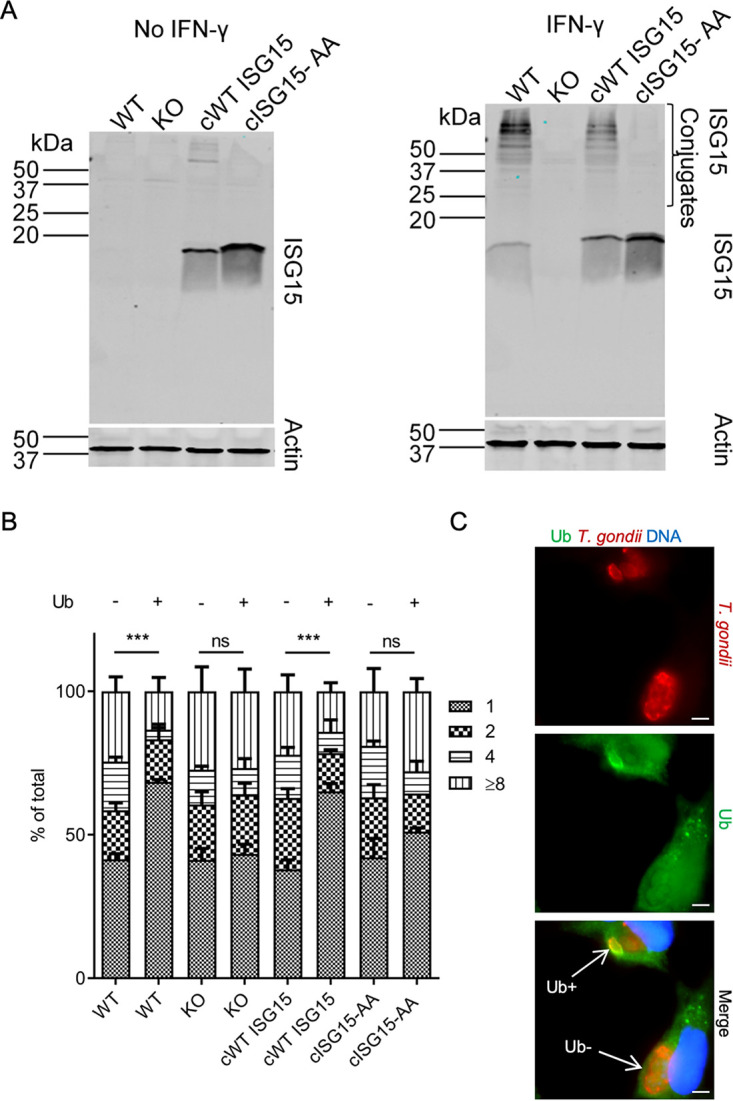
ISG15 is required to restrict T. gondii growth in IFN-γ-activated A549 cells. (A) Immunoblot of ISG15KO cells complemented with functional wild-type ISG15 (cWT ISG15) or ISGylation-defective ISG15 (cISG15-AA) construct. Cells were grown with or without IFN-γ (100 U/ml) for 24 h. ISG15 and ISGylation levels in cell lysates were determined by immunoblotting with rabbit polyclonal ISG15 antibody followed by LI-COR IRDye 800CW (green) goat anti-rabbit IgG. Actin was probed as the loading control using mouse monoclonal actin antibody and LI-COR IRDye 680RD (red) goat anti-mouse IgG. (B and, C) Wild-type (WT), ISG15 KO, and complemented (cISG15 or cISG15-AA) A549 cells were cultured in IFN-γ (100 U/ml) for 24 h and infected with type III T. gondii (CTG). Cells were washed 2 h postinfection, and the numbers of parasites per vacuole were counted in ubiquitin-positive (Ub+) versus ubiquitin-negative (Ub-) vacuoles at 36 h postinfection by immunofluorescence microscopy. The values represent means ± standard error from three independent experiments. Two-way ANOVA was used to determine statistical significance. *P* values of ≤0.001 (***) were considered statistically significant. ns, not significant. Representative images are shown on the right. Scale bar = 5 μm. Parasites (red) were stained with rabbit anti-GRA7. Ubiquitin (green) was stained with mouse monoclonal ubiquitin antibody. Alexa Fluor 594 (red) conjugated rabbit and Alexa Fluor 488 (green) conjugated mouse IgG antibodies were used as secondary antibodies for staining parasites and ubiquitin, respectively. Nuclei were stained with DAPI. The white arrows indicate parasite-containing, Ub-positive (Ub+), and Ub-negative (Ub-) vacuoles.

The studies described above indicate that ISG15 is important for recruitment of autophagy mediators to the PV surrounding T. gondii. To determine whether ISG15 plays a functional role in this pathway, we compared the growth of T. gondii in wild-type and ISG15 KO cells. To distinguish between conjugation-dependent and -independent functions, we used the ubiquitin C promoter to generate complemented wild-type ISG15 (referred to as cWT ISG15) or mutant ISG15 cell lines, which cannot be conjugated due to mutation of critical glycine residues that are normally involved in conjugation (50) (referred to as cISG15-AA) (Fig. 5A). Despite constitutive expression of cWT ISG15 and cISG15-AA in complemented lines, we only observed minor ISGylation in the absence of IFN-γ (Fig. 5A). However, IFN-γ treatment resulted in enhanced levels of ISGylated proteins in complemented cWT ISG15 cell lysates, while this enhancement was absent in complemented mutant cISG15-AA cell lysates (Fig. 5A).

To examine the role of ISG15 in IFN-γ-dependent growth restriction, we stimulated wild-type, ISG15 KO, complemented wild-type ISG15 (cWT ISG15), or complemented mutant ISG15 (cISG15-AA) cells with IFN-γ and measured T. gondii growth 36 h postinfection. Growth of the parasite normally proceeds by binary fission with a half-life of ∼8 h such that at 36 h postinfection, the vacuoles contain clusters of parasites that range from 1 parasite/vacuole to 8 parasites/vacuole. Previous studies have shown that vacuoles that become ubiquitinated in IFN-γ-treated HeLa cells are restricted in growth such that this distribution is skewed to smaller cluster sizes (i.e., replication is inhibited) (22). The growth inhibition phenotype depends on ubiquitination and is not evident in Ub-negative compartments (22). As such, we scored the number of parasite/ubiquitin (Ub)-positive vacuoles at 36 h postinfection in IFN-γ-treated cells. Wild-type (WT) cells restrict the growth of T. gondii in Ub-positive vacuoles, as shown by the larger number of single-parasite vacuoles, and this phenotype was lost in the ISG15 KO, where vacuole sizes returned to the broader distribution seen in wild-type parasites that were not ubiquitinated (Fig. 5B and C). Complementation of ISG15 KO cells with the wild-type ISG15 construct (i.e., cWT ISG15) rescued the growth restriction phenotype, while complementation with the ISGylation-defective construct (i.e., cISG15-AA) failed to rescue growth restriction (Fig. 5B). None of the lines showed significant differences in parasite growth in ubiquitin-negative vacuoles, consistent with previous findings in HeLa cells. We conclude that ISG15 and ISGylation are important for IFN-γ-dependent restriction of T. gondii by the noncanonical autophagy pathway in human cells.

### ISG15 establishes cross talk between IFN-γ and ATG pathways.

**FIG 6:**
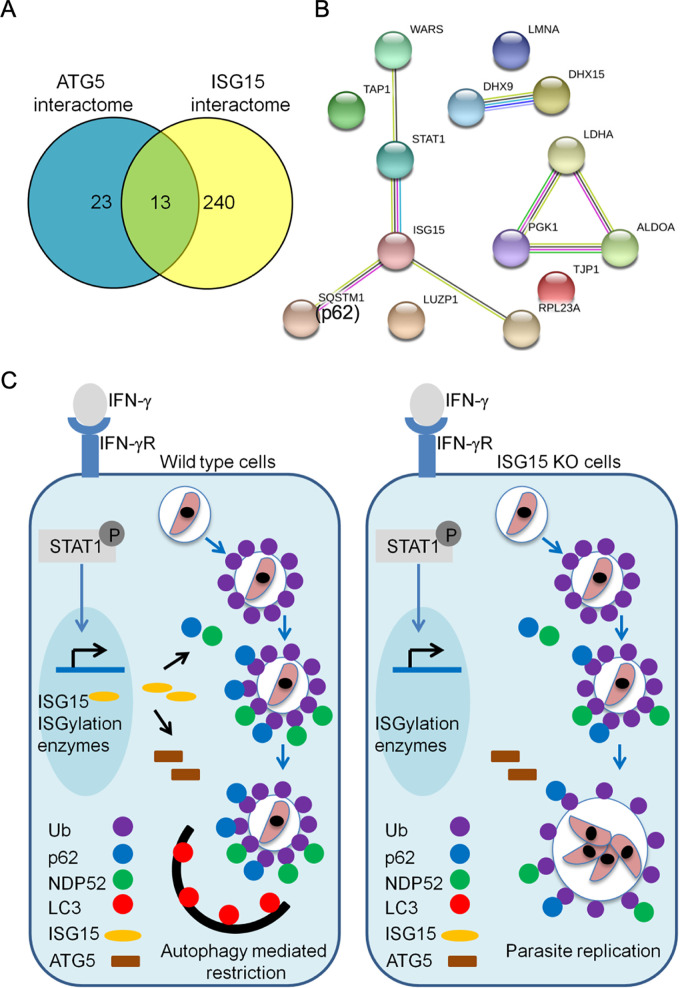
Interaction of ISG15 and ATG5 in restriction of T. gondii growth in human cells. (A) Venn diagram representing the overlap between BirA-ATG5 proximity labeling and proteins immunoprecipitated with ISG15. Proteins identified by mass spectrometry from ISG15 immunoprecipitation assay were analyzed using Scaffold version 4. 0. The normalized weighted spectra of IFN-γ-stimulated cWT ISG15 samples were compared with conjugation-defective cISG15-AA samples. Proteins were included based on peptide count (≥3 in cWT ISG15) and fold change in normalized weighted spectra (≥1) (Table 2). (B) The ISG15 interactome was built in the STRING database version 10.5. Nodes (filled circles) represent proteins and edges indicate interactions as described in Fig. 1 (C) Proposed model for role of ISG15 in connecting autophagy and IFN-γ-dependent restriction of T. gondii growth in human cells.

ISG15 lies at the center of the ATG5 interactome, and it is predicted to interact with a number of host defense proteins that are present in the interactome. To establish the central role of ISG15 in this network, we immunoprecipitated ISG15 from IFN-γ-treated A549 cells complemented with wild-type ISG15 (i.e., cWT ISG15) and the ISGylation-defective ISG15 form (i.e., cISG15-AA). Mass spectrometry results from three independent replicates were used to characterize interacting proteins (based on ≥3 peptides in a cWT ISG15 sample and fold change in normalized weighted spectra of ≥1 in wild-type versus mutant ISG15) that were either conjugation dependent or independent (Table S1). Comparing all ISG15 immunoprecipitated proteins to the ATG5 proximity labeling interactome, we identified 13 shared proteins (Table 1 and 2, Fig. 6A). The interaction of 10 of these shared proteins is partially conjugation dependent, as their fold change in normalized weighted spectra was >1 in wild-type (i.e., cWT ISG15) relative to mutant ISG15 (i.e., cISG15-AA) samples (Table 2). The STRING network of the 13 shared proteins showed interactions similar to the ATG5 interactome and included proteins involved in IFN-γ responses (i.e., STAT1, WARS, and TAP1), glycolytic enzymes (i.e., ALODOA, LDHA, and PGK1), and several RNA binding factors important in antiviral defense (i.e., DHX9 and DHX15) (Fig. 1C and 6B). Interestingly, the adaptor protein p62 (SQSTM1), which showed defective recruitment to PV in the absence of ISG15 (Fig. 1B), was 1.5-fold more abundant in wild-type ISG15 complemented cells (cWT ISG15) compared to mutant ISG15 complemented cells (cISG15-AA), suggesting that its interaction is partially conjugation dependent (Table 2). The adaptor NDP52 was not abundant in either sample, suggesting that its interaction with ISG15 is of lower affinity, although it was observed in one of the cWT ISG15 samples (two peptides in one of the three replicates).

**TABLE 2:** Overlap between ISG15 immunoprecipitation and BirA-ATG5 interactome

Identified proteins	Gene name	Fold change^*a*^	cISG15-AA^*b*^	cWT ISG15^*b*^
Fructose-bisphosphate aldolase A	ALDOA	6.3	2	13
Leucine zipper protein 1	LUZP1	6	1	6
Tight junction protein 1 (Zona occludens 1), isoform CRA_a	TJP1	3.4	2	8
Prelamin-A/C	LMNA	2.4	5	12
l-lactate dehydrogenase A chain	LDHA	2.2	5	12
Signal transducer and activator of transcription 1-alpha/beta	STAT1	1.8	7	13
Sequestosome-1	SQSTM1	1.5	10	15
Phosphoglycerate kinase 1	PGK1	1.3	3	4
ATP-dependent RNA helicase A	DHX9	1.1	23	24
Pre-mRNA-splicing factor ATP-dependent RNA helicase	DHX15	1.1	4	5
60S ribosomal protein L23a	RPL23A	1	11	11
Tryptophan-tRNA ligase, cytoplasmic	WARS	1	8	8
Antigen peptide transporter 1	TAP1	1	5	5

a Fold change in normalized weighted spectra in cWT ISG15 versus cISG15-AA.

b Columns represent normalized weighted spectra over 3 replicates.

10.1128/mBio.00852-20.4 TABLE S1 Proteins identified in ISG15 immunoprecipitation. Download Table S1, XLS file, 0.04 MB. Copyright © 2020 Bhushan et al. 2020 Bhushan et al. This content is distributed under the terms of the Creative Commons Attribution 4.0 International license.

## DISCUSSION

Control of T. gondii replication in human HeLa cells relies on a noncanonical ATG pathway that is dependent on IFN-γ (22). Ablation of core members of this pathway, such as ATG16L1 or ATG7 compromises IFN-γ-mediated control of parasite replication (22). Concordant with this dependence, recruitment of ATG adaptors and LC3B to vacuoles containing susceptible parasites requires activation by IFN-γ (22). However, the basis for why IFN-γ control of infection requires components of the ATG pathway has not been defined in previous studies. Here, we demonstrate that ISG15, which is upregulated by IFN-γ along with other members of the ISGylation pathway, interacts with members of the ATG pathway. ATG mediators p62, NDP52, and LC3 showed decreased recruitment to vacuoles containing a susceptible strain of the parasite in the absence of ISG15, despite there being no global impact on the ATG pathway. ISG15 was required for control of parasite replication in IFN-γ-treated cells as shown by loss of control in ISG15 knockout cells. Moreover, ISGylation of cellular proteins was important for ATG-mediated cell-autonomous immunity against T. gondii. Collectively, our data establish a role of ISG15 in linking the ATG pathway with IFN-γ-dependent restriction of T. gondii growth in human cells.

To define potential interactions between ATG and IFN-γ, we used proximity-dependent biotin labeling to identify partners of ATG5 that were induced by IFN-γ treatment. Network analysis of the ATG5 interactome identified ISG15 as a central hub connecting ATG proteins with IFN-γ-induced genes, many of which are involved in pathogen control (9, 25, 26). ISG15 was strongly induced in IFN-γ-stimulated samples and showed greater association with ATG5 relative to control cells. Enhanced IFN-γ-dependent ISGylation in human cell lines demonstrated that IFN-γ treatment also upregulates enzymes of the ISGylation pathway, as described previously for type I IFN (51). ISG15 expression is normally induced in host cells by a variety of factors, including viral infection, TLR ligands such as lipopolysaccharide and poly IC, tumor necrosis factor-α, or type I, II, and III IFNs (29, 48, 52). ISG15 is best known for its role in viral infection (53), and in contrast, there are only a few studies describing its role in bacterial and fungal infections (54–58). Our data indicate that ISG15 also plays an important role in IFN-γ-dependent cell-autonomous immunity against the protozoan parasite T. gondii in human cells. Type I IFN also contributes to cell-autonomous control of T. gondii infection (59), suggesting that ISG15 may also play an important role in this context. Separate studies have shown that in cells where ISGylation is enhanced, induction of autophagy is associated with greater control of intracellular Listeria monocytogenes (60). Together, these findings suggest that ISG15 plays a broader role in control of intracellular pathogens than previously recognized from its role in antiviral defenses.

Our findings demonstrate that ISG15 promotes IFN-γ-dependent recruitment of ATG mediators p62, NDP52, and LC3 to PV. Each of these adaptors was decreased, although not completely absent, in ISG15 KO cells, suggesting that ISG15 facilitates their recruitment to the PV. Interestingly, ubiquitination of the PV did not require ISG15, suggesting that this modification lies upstream of the recruitment of ATG adaptors. Kinetic studies of the labeling of PVs containing susceptible strains of T. gondii in HeLa cells support ubiquitination as the initial step in the process, followed by recruitment of p62 and NDP52 and, finally, deposition of LC3 (22). The kinetics of recruitment observed here in A549 cells are also consistent with this pathway of sequential recruitment. Vacuole ubiquitination is also an early step in the process of xenophagy, a pathway responsible for clearance of intracellular bacteria (23). Intracellular vacuoles containing pathogens such as *Salmonella* and *Mycobacterium* often become damaged and reveal glycans on the inner surface of the vacuole that are recognized by the cytosolic protein galectin 8, which is recognized by NDP52 through a GALB1 domain (61). Subsequently, a variety of ubiquitin ligases, including LRSAM1, PARKIN, and LUBAC, ubiquitinate intracellular bacteria, leading to clearance by xenophagy (62). Studies of T. gondii PVs formed in HUVEC cells stimulated with IFN-γ also demonstrated slightly elevated levels of galectin 8 staining, and these compartments are also ubiquitinated and recruit ATG adaptors such as NDP52 and p62 (24). Whether early membrane damage and galectin staining also underlie susceptibility of T. gondii PVs to ubiquitination in HeLa cells remains unknown. There are more than 600 putative ubiquitin ligases in human cells (63), and the basis for selective ubiquitination of PV containing T. gondii is presently unknown. However, type I strains are highly resistant to this pathway (22), suggesting the presence of parasite factors that are able to counteract ATG-mediated control.

Previously, it was reported that bone marrow-derived macrophages from ISG15-deficient mice have reduced levels of ATG proteins on stimulation with type I IFN (64). However, in our studies using the human lung epithelial cell line A549, we did not observe decreased expression of ATG proteins in the absence of ISG15. Although previous reports have indicated that T. gondii infection can counteract autophagy to promote parasite survival (65), we did not observe appreciable differences in the core components involved in restriction of parasite growth in response to IFN-γ treatment. Additionally, macroautophagy induced by a combination of rapamycin and bafilomycin A1 was not affected in A549 cells in the absence of ISG15. Hence, ISG15 does not appear to be required for macroautophagy, but rather, it specifically affects recruitment of ATG adaptors such as NDP52, p62, and LC3 to the PV. The mechanism by which ISG15 facilitates recruitment of these adaptors is not known, but it may rely on structural similarity between ubiquitin and ISG15. The adaptor p62 has a ubiquitin binding domain (UBA) that preferentially recognizes K63-linked ubiquitin (37). Additionally, p62 may interact with ISG15 due to its structural resemblance to ubiquitin, a feature that has previously been shown to facilitate recruitment of proteins to aggresomes for degradation (66). Similarly, the presence of a ubiquitin binding domain (UBZ) in NDP52 (67) may facilitate interaction with ISG15 and recruitment to the PV. In addition to the potential for these interactions, our data also argue that the conjugation-dependent functions of ISG15 are primarily responsible for ATG-mediated control of T. gondii growth. Consistent with this prediction, the adaptor p62 was also detected as a conjugation-dependent interaction, suggesting that it can be covalently modified by ISG15, which may further enhance recruitment. However, recruitment of these adaptors to PV is not completely abrogated in the absence of ISG15, suggesting that other mediators may also be involved.

In a previous study using HeLa cells, we demonstrated that recruitment of GFP-LC3B was associated with restricted growth of the parasite in HeLa cells (22). Here, we used an antibody to LC3, which reportedly recognizes LC3A, LC3B, and LC3C but not GABARAP or GATE-16, to show recruitment to PVs in A549 cells. This antibody also cross-reacts with mouse LC3 isoforms, and it has been used to monitor recruitment of LC3 to PVs containing susceptible strains of T. gondii in mouse MEFS activated with IFN-γ (20). Although LC3B is commonly used as a marker for autophagosomes, human cells express three isoforms of LC3 (i.e., LC3A, LC3B, and LC3C) and three related genes (i.e., GABARAP, GABARAPL1, and GATE-16 [also known as GABARAPL2]), while mice express five related proteins (LC3a, LC3b, Gabarap, Gabarapl1, and Gate-16) (68, 69). Previous studies in the murine system demonstrated that Gate-16 is the primary isoform associated with clearance of T. gondii in IFN-γ-activated MEF cells. These findings suggest that evaluating other members of this family might be informative for understanding the role of ATG in controlling growth of T. gondii in IFN-γ-activated human cells.

ISG15 can exert its functional role as either free or conjugated forms (29, 70). In the present study, we show that ISGylation is important for IFN-γ-dependent restriction of parasite by ATG in human cells. The loss of IFN-γ-dependent parasite growth restriction in ISG15 KO cells was complemented by reexpression of a wild-type copy of the protein, but not by a conjugation-defective form that lacks key glycine residues required for conjugation (50). Our result is in contrast to the studies in mice where unconjugated ISG15 was found to contribute to formation of IL-1β-producing dendritic cells during T. gondii infection (30). ISG15 knockout mice showed a minor survival advantage at a low dose of infection and no difference in parasite load, while at a higher dose, similar survival was observed (30). The absence of a major phenotype for SG15 knockout mice infected with T. gondii is likely due to the different pathways that are important for cell-autonomous control in murine and human cells. In murine cells, recruitment of IRGs and GBPs requires a noncanonical autophagy pathway (15); however, there is no known role for ISG15 in this critical pathway. In contrast, human cells use a wider variety of mechanisms (71), including a noncanonical pathway for vacuole engulfment and growth restriction that exists in HeLa cells (22), and we show in the present studies in A549 cells. Our findings indicate that the latter pathway is partially dependent on ISG15, further distinguishing the mechanisms that operate in human cells. Our findings provide a further example of species-specific differences in the functions of ISG15, which have been extensively characterized previously in antiviral responses (72–74).

Our proteomic analysis demonstrates considerable overlap between proteins that interact with ATG5, as defined by proximity labeling, and proteins that immunoprecipitate with ISG15. A large percentage of the shared proteins are also induced by IFN-γ, establishing the central role of ISG15 connecting the ATG pathway with IFN-γ-dependent control of T. gondii in human cells. We show that ISG15 interacts with >200 proteins in a conjugation-dependent and -independent manner following stimulation with IFN-γ. Our data are consistent with previous results showing that ISG15 interacts with >300 proteins in the presence of type I IFN (52). It is likely that IFN-γ-dependent protein modification by ISG15 can affect diverse cellular pathways and may have a broader role besides governing cell-autonomous immunity to T. gondii.

In conclusion, our data identify the role of ISG15 in autophagy and IFN-γ-dependent control of T. gondii infection in human cells as depicted in Fig. 6C. ISG15 recruits the adaptors p62 and NDP52 to the PV, which in turn results in deposition of LC3, leading to growth restriction of T. gondii by ATG. Interestingly, the interaction of p62 was largely conjugation dependent, while NDP52 showed a weak conjugation-dependent phenotype. In either case, the absence of ISG15 impaired adaptor recruitment to the PV and resulted in failure of parasite restriction. We conclude that ISG15 connects the ATG and IFN-γ-dependent control of T. gondii infection in human cells, suggesting that ISG15 may also contribute to the control of other infections where IFN-γ-induced autophagy plays an important role (75–77).

## MATERIALS AND METHODS

### Antibodies and reagents.

Streptavidin magnetic beads were obtained from Thermo Fischer Scientific. Streptavidin conjugated to IRDye 800CW (LI-COR Biosciences) was used for detection of biotinylated proteins on blots. ISG15 was detected using rabbit polyclonal ISG15 antibody (15981-1-AP; Proteintech). Actin was detected using mouse anti-actin clone C4 (MAB1501; Millipore). Ubiquitin was detected using mouse antibody clone FK2 (04-263; EMD Millipore Corporation). p62 was detected using guinea pig polyclonal p62 antibody (GP62-C; Progen). NDP52 was detected with rabbit polyclonal antibody (12229-1-AP; Proteintech). LC3 (immunofluorescence assay) was detected with rabbit polyclonal antibody (PM036; MBL International Corporation). Rabbit polyclonal LC3 (NB100-2220) and monoclonal rabbit ATG5 (12994S), antibodies for immunoblotting, were obtained from Novus Biologicals and Cell Signaling Technology, respectively. Antibodies used for detecting T. gondii parasites (mouse monoclonal DG52 against SAG1 and rabbit polyclonal anti-whole tachyzoite lysate) or the parasitophorous vacuole (rabbit polyclonal GRA7) have been previously described (22). Goat anti-mouse, goat anti-rabbit, and goat anti-guinea pig secondary antibodies conjugated to Alexa Fluor 488, Alexa Fluor 594, or Alexa Fluor 647 (Life Technologies) were used for immunofluorescence. Goat anti-mouse, anti-guinea pig, or anti-rabbit IgG labeled with IRDye 800CW or 680RD (LI-COR Biosciences) were used as secondary antibodies for immunoblotting.

### Parasite culture.

Tachyzoites of the type III CTG strain (ATCC 50842) were cultured in human foreskin fibroblast cells grown in Dulbecco’s modified Eagle’s medium (Invitrogen) supplemented with 3% fetal bovine serum (FBS) (GE Healthcare Life Sciences), 10 mM glutamine (Thermo Fisher Scientific), 10 mM HEPES (Sigma-Aldrich), pH 7.5, and 10 μg/ml gentamicin (Thermo Fisher Scientific) at 37°C in 5% CO_2_. Naturally egressed parasites were harvested as described previously (22). Parasite cultures and host cell lines were confirmed as negative for mycoplasma using a e-Myco plus kit (Intron Biotechnology).

### Cell culture.

HeLa cells stably expressing promiscuous biotin ligase BirA-ATG5 as a fusion protein were generated by lentiviral transduction. BirA was cloned in-frame with ATG5 into the NotI restriction site in pCDH-MCS-T2A-Puro (CD522A-1; System Biosciences) using the In-Fusion HD cloning kit (TaKaRa) and confirmed by Sanger sequencing. Lentivirus was produced in HEK293T cells by cotransfecting BirA-ATG5 expression plasmid, envelope plasmid pCMV-VSVG (8454; Addgene), and packaging plasmid psPAX2 (12260; Addgene). HeLa cells were transduced with the lentivirus and maintained in medium supplemented with 1 μg/ml puromycin (Thermo Fisher Scientific). ATG16L1 knockout cells have been previously described (22). HeLa cells were grown in minimum essential medium (Sigma-Aldrich) supplemented with 10% defined fetal bovine serum (FBS; GE Healthcare Life Sciences), 4 mM l-glutamine (Thermo Fisher Scientific), and 10 mM HEPES (Sigma-Aldrich), pH 7.5, at 37°C in 5% CO_2_.

Wild-type and ISG15 knockout (ISG15KO) A549 cells were produced with a CRISPR/Cas9 approach. Target sequences for CRISPR interference were selected using the CRISPR guide design tools available at the Zhang laboratory (https://zlab.bio/guide-design-resources). Two seed sequences preceding the protospacer-adjacent motif (PAM) used are as follows: g5, CGACGAACCTCTGAGCATCC; g6, GCACGCCTTCCAGCAGCGTC. The sense and antisense oligonucleotides were designed to incorporate the restriction enzyme site BbsI of pX459 (62988; Addgene) bicistronic expression vector expressing Cas9 and synthetic single-guide RNA (78). The oligonucleotide DNAs were annealed, phosphorylated, and incorporated into pX459 vector linearized with BbsI restriction enzyme. A549 cells were transfected with ISG15-targeting pX459 plasmid with Lipofectamine 2000 (Thermo Fisher Scientific) according to the manufacturer’s instructions. The cells were selected with puromycin (2 μg/ml for 48 h) and replated to a 96-well plate at a density of 0.5 cells per well. The individual colonies were picked, and ISG15 expression was checked by immunoblotting. The deletion of ISG15 loci in the genome was further confirmed by targeted sequencing (Genome Engineering Center, Washington University). The percentage of respective indels and the sequencing results are shown in the supplemental material (Fig. S3A and B). Wild-type and ISG15 KO A549 cells were grown in Dulbecco’s modified Eagle’s medium supplemented with 10% FBS, 10 mM glutamine, 10 mM HEPES, pH 7.5, and 10 μg/ml gentamicin (Thermo Fisher Scientific) at 37°C in 5% CO_2_.

10.1128/mBio.00852-20.3 FIG S3 Target sequencing confirmed the deletion of the ISG15 gene in A549 cells. (A) Validation of deletion of the sgRNA targeted area and percentage of each indel in A549 ISG15KO cells. (B) Alignment of indels to the ISG15 coding sequence to confirm deletion mediated by the CRISPR/Cas9 approach. Download FIG S3, TIF file, 2.2 MB. Copyright © 2020 Bhushan et al. 2020 Bhushan et al. This content is distributed under the terms of the Creative Commons Attribution 4.0 International license.

### Complementation of A549 cells.

ISG15 KO A549 cells were transduced with lentivirus to express complemented copies of wild-type ISG15 (cWT ISG15) and ISGylation-defective ISG15 (cISG15-AA). To generate a conjugation-defective form of ISG15, the glycines that function in conjugation of the wild-type sequence LRLRGG were replaced by alanine to generate a construct expressing LRLRAA (50). The ISGylation-defective ISG15 (cISG15-AA) was constructed by changing the nucleotides corresponding to glycine (GGT GGG) residues to those encoding alanine (GCG GCG) in the reverse primer. HA-tagged full-length wild-type ISG15 and its ISGylation-defective form were cloned in lentivirus vector pCDH-UbC-MCS-EF1α-Hygro (CD615B-1; System Biosciences) at the NheI and NotI sites using the In-Fusion HD cloning kit (TaKaRa). Lentivirus was produced by cotransfecting lentiviral expression vector, pCMV-VSVG (8454; Addgene), and psPAX2 (12260; Addgene) in HEK293T cells. Virus-containing cell supernatants were collected 48 h posttransfection, filtered through a 0.45-μm filter, and used to transduce A549 ISG15 KO cells. Cells were grown in the presence of 1 mg/ml hygromycin B (GoldBio) 48 h postransduction to select for stably integrated cells.

### Sample preparation for mass spectrometry and data analysis.

**Permissive biotin labeling.** HeLa cells expressing BirA-ATG5 were cultured with or without 100 U/ml IFN-γ (R&D Systems) for 24 h and grown in the presence or absence of biotin (Sigma-Aldrich) for 18 h. Proteins were extracted in radioimmunoprecipitation assay (RIPA) buffer (50 mM Tris, pH 7.5), 150 mM sodium chloride, 0.1% sodium dodecyl sulfate, 0.5% sodium deoxycholate, and 1% Triton X-100) containing EDTA-free protease inhibitor cocktail (Roche). The extracted proteins were affinity purified by incubation with streptavidin magnetic beads (Thermo Fisher Scientific). At the end of the incubation period, beads were washed as described previously (79) and given a final wash with PBS.

**Immunoprecipitation.** For immunoprecipitation, lysates were prepared from A549 cells and were incubated with rabbit polyclonal ISG15 antibody (Proteintech) for 16 h at 4°C. The immunocomplex was pulled down with Dynabeads protein G (Thermo Fisher Scientific). The beads were separated and washed thrice with PBS-Tween (0.02%) and thrice with PBS.

**Mass spectrometry (MS) analysis.** The beads were submitted for analysis to the Proteomics and Metabolomics Facility, Center for Biotechnology, University of Nebraska Lincoln. The magnetic bead samples were resuspended in ammonium bicarbonate and reduced with 5 mM DTT at 37°C for 1 h. The proteins were alkylated (10 mM iodoacetamide for 20 min at 22°C in the dark), followed by quenching with DTT (1 M). Trypsin digestion was done overnight at 37°C with shaking. The supernatant was dried and redissolved in 2.5% acetonitrile and 0.1% formic acid. Samples were run by nanoscale liquid chromatography (nanoLC)-MS/MS using a 2-h gradient on a 0.075-mm by 250-mm Waters CSH C_18_ column feeding into a Q-Exactive high-frequency (HF) mass spectrometer. Data were analyzed using Mascot version 2.6.2 (Matrix Science, London, UK). Mascot was set up to search the cRAP_20150130.fasta (123 entries), Custom5_20190917.fasta (1 entry), and UniProt-human_20190219 database (73,928 entries) assuming the digestion enzyme trypsin. Mascot was searched with a fragment ion mass tolerance of 0.060 Da and a parent ion tolerance of 10.0 ppm. Deamidation of asparagine and glutamine, oxidation of methionine, and carbamidomethylation of cysteine were specified in Mascot as variable modifications.

**Network analysis.** For both sets of samples, identified proteins were analyzed using Scaffold version 4.0 (number of peptides, 2; protein threshold, 99%; peptide threshold, 95%) (80). The interaction among the shortlisted proteins was generated online using the STRING database at https://version-10-5.string-db.org/cgi/input.pl (81) using a minimum interaction score of 0.4.

### Western blotting.

Beads or cell lysates were boiled in SDS sample buffer, resolved on polyacrylamide gel, transferred to the nitrocellulose membrane, and probed with IRDye conjugated streptavidin or primary antibodies against cellular proteins followed by incubation with IRDye conjugated secondary antibodies.

### Immunofluorescence microscopy.

Samples were viewed under a Zeiss Axioskop 2 MOT Plus microscope (Carl Zeiss) using a ×63 Plan-Apochromat lens, numerical aperture 1.40, and images were acquired with an AxioCam MRm camera (Carl Zeiss) with AxioVision version 4.6. Images were processed in Fiji-ImageJ and Photoshop CS6 version 13.0x64.

### Recruitment of adaptors and parasite vacuolar growth.

A549 cells were stimulated with IFN-γ (100 U/ml) for 24 h, infected with tachyzoites of the type III T. gondii strain CTG, and washed 2 h postinfection to remove extracellular parasites. The cells were fixed in 4% formaldehyde 6 h postinfection to study the recruitment of autophagy mediators to the parasite by using antigen-specific antibodies. Control cells were grown in the absence of IFN-γ. T. gondii was localized using antibodies against the tachyzoites or parasite surface antigen 1 (SAG1) or parasite dense granule protein (GRA7) present on the PV. Alternatively, for examining the colocalization of ubiquitin and adaptors on the PV, we infected cells with a green fluorescent protein (GFP)-expressing strain of CTG. Recruitment of ubiquitin, p62, NDP52, and LC3 were quantified from 30 or more PV on each of three separate coverslips per group. Vacuolar growth of T. gondii was studied 36 h postinfection by enumerating the number of parasites per vacuole from 30 PV on 3 individual coverslips.

### Autophagosome formation.

A549 cells were grown with or without rapamycin 5 μM (Sigma-Aldrich) for 24 h and then treated with bafilomycin A1 (100 nm) (InvivoGen) for 4 h before fixation in 4% formaldehyde. Autophagosome was detected using rabbit anti-LC3 staining to enumerate LC3 puncta from 30 or more cells on each of 3 separate coverslips per group. LC3 puncta per cell were quantified using Volocity version 6.3 software.

### Statistical analysis.

Statistical analyses were done using GraphPad Prism 7. Unless otherwise specified, data from three independent experiments, each with three internal replicates, were combined and plotted as means ± standard error of means. Two-way analysis of variance (ANOVA) was used to determine statistical significance between samples, where the first variable was the groups and the second variable was separate experiments. *P* values of ≤0.05 (*), ≤0.01 (**), ≤0.001 (***), and ≤0.0001 (****) were considered statistically significant.
