# Supplementary figures and images for: ISG15 Connects Autophagy and IFN-γ-Dependent Control of Toxoplasma gondii Infection in Human Cells
Source: mBio. 2020 Oct 6;11(5):e00852-20. doi: 10.1128/mBio.00852-20 (PMC7542356; doi:10.1128/mBio.00852-20)

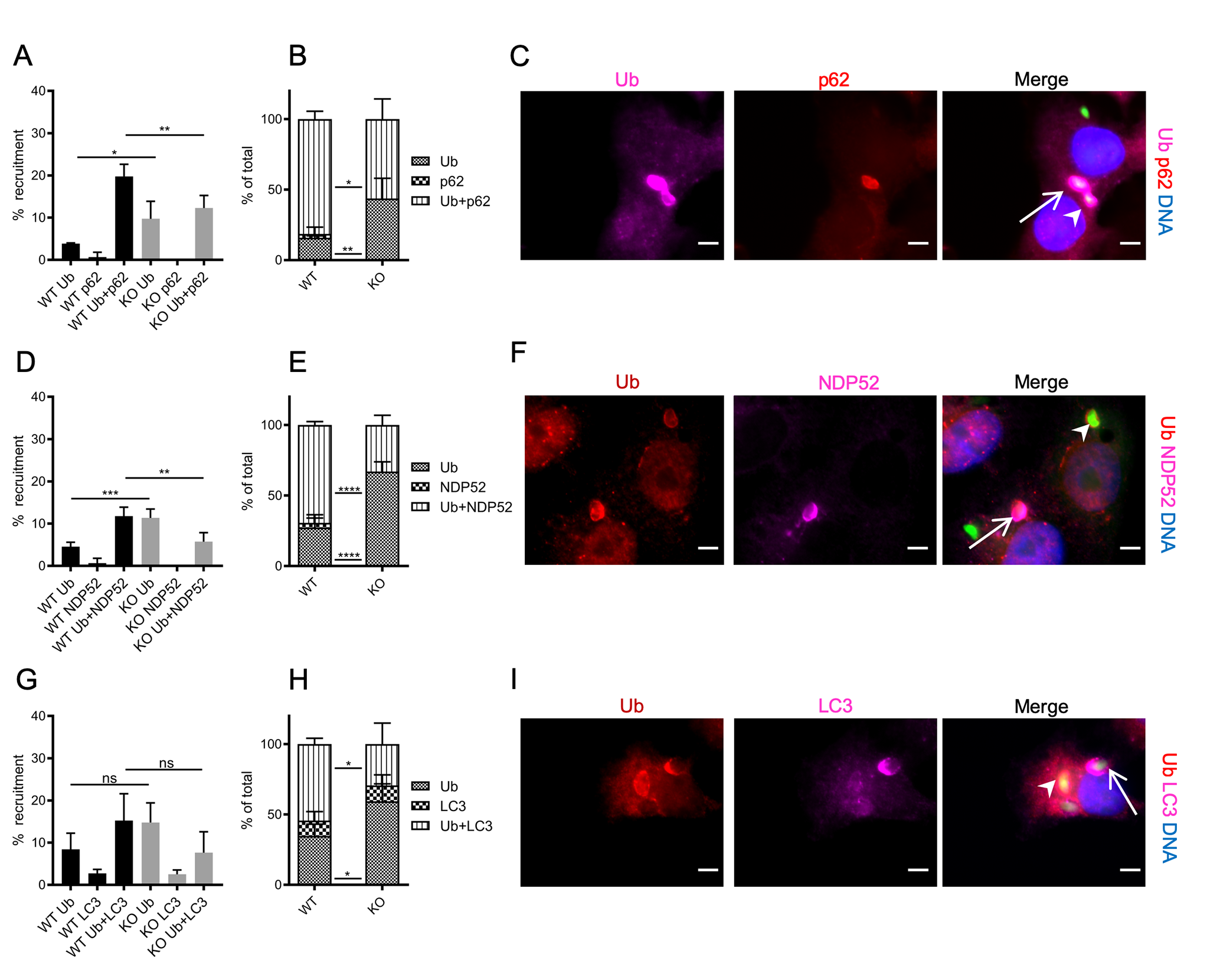

Supplement: FIG S1 [file mBio.00852-20-sf001.tif]

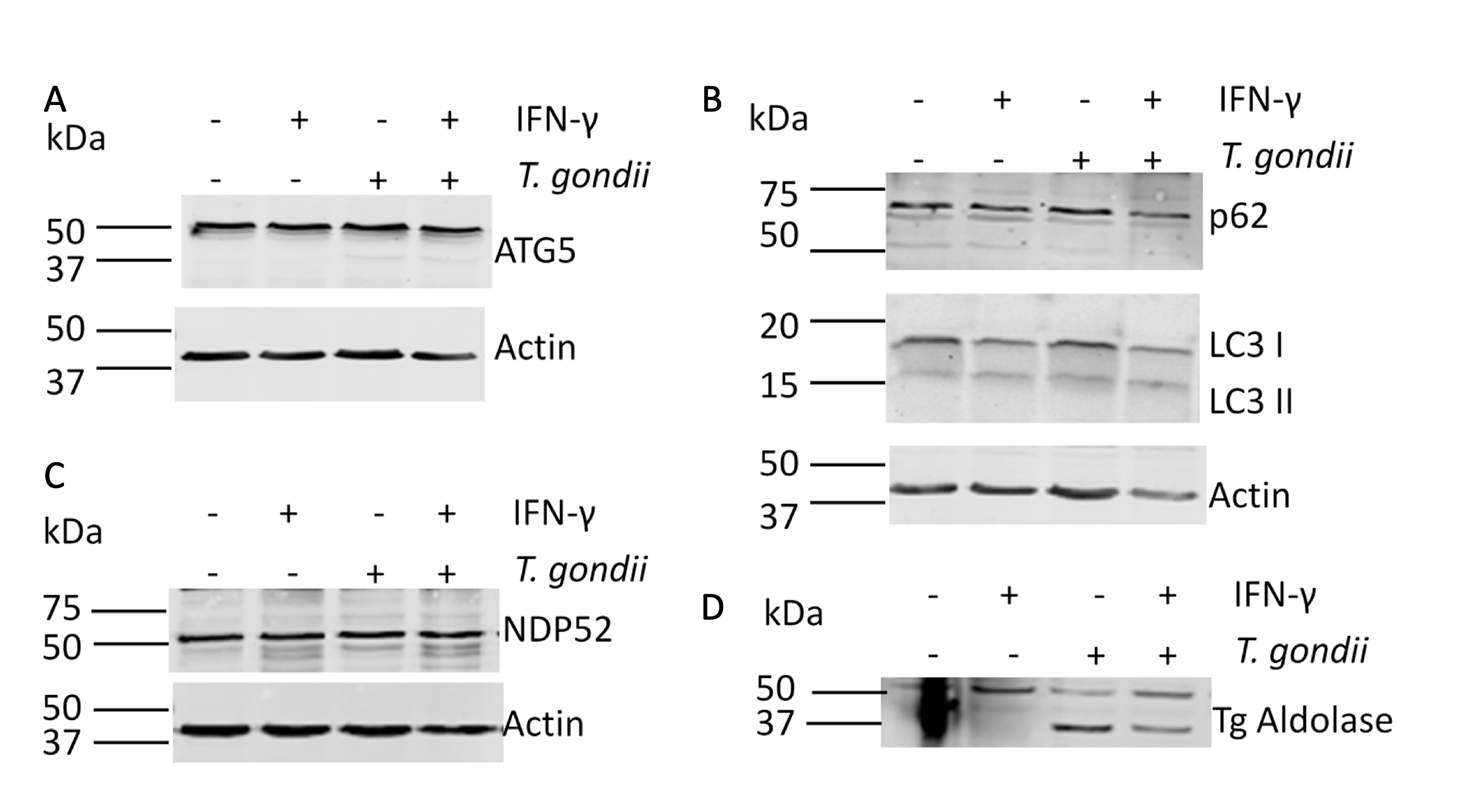

Supplement: FIG S2 [file mBio.00852-20-sf002.tif]
